# The Association between Perceived Adequacy and Capacity for School Food Policy Implementation with Food Availability and Policy Adherence in Nova Scotia, Canada

**DOI:** 10.3390/ijerph16111974

**Published:** 2019-06-04

**Authors:** Jessie-Lee D. McIsaac, Tarra L. Penney, Louise Mâsse, Sara F.L. Kirk

**Affiliations:** 1 Healthy Populations Institute, Dalhousie University, PO Box 15000, Halifax, NS B3H 4R2, Canada; Jessie-Lee.McIsaac@msvu.ca; 2 Faculty of Education, Mount Saint Vincent University, 166 Bedford Highway, Halifax, NS B3M 2J6, Canada; 3 MRC Epidemiology Unit/CEDAR, University of Cambridge, Cambridge CB2 0SL, UK; tarra.penney@mrc-epid.cam.ac.uk; 4 School of Population and Public Health, Faculty of Medicine, University of British Columbia, Vancouver, BC V6T 1Z3, Canada; lmasse@bcchr.ubc.ca; 5 School of Health and Human Performance, Faculty of Health, Dalhousie University, PO Box 15000, Halifax, NS B3H 4R2, Canada

**Keywords:** school health, child/adolescent health, health education, health promotion, school nutrition, school food, policy

## Abstract

Supporting the implementation of school food and nutrition policies is an international priority to encourage healthier eating among children and youth. Schools are an important intervention setting to promote childhood nutrition, and many jurisdictions have adopted policies, guidelines, and programs to modify the school nutrition environment and promote healthier eating. The purpose of this study was to explore the association between perceived adequacy of facilities or equipment and capacity of staff to support policy implementation with food availability and policy adherence in the province of Nova Scotia (NS), Canada, one of the first regions in Canada to launch a comprehensive school food and nutrition policy (SFNP). A cross-sectional online survey was conducted in 2014–2015 to provide a current-state assessment of policy implementation and adherence. Adequacy and capacity for food policy implementation was used to assess policy adherence through the availability of prohibited ‘minimum’ nutrition foods. An exploratory factor analysis was conducted on a selection of available foods, and ‘slow’- and ‘quick’-service food composition measures were dichotomized for food availability. Schools with above-average perceived adequacy and capacity for policy implementation had greater odds (OR = 3.62, CI = 1.56, 8.40) of adhering to a lunch policy, while schools that adhered to a snack and lunch policy had lower odds (OR = 0.48, CI = 0.23, 1.01 and OR = 0.18, CI = 0.08, 0.41) of serving quick-service foods. This study identified the need for appropriate adequacy of facilities or equipment and capacity of staff for policy implementation to ensure policy adherence and improve the school food environment. These findings highlight the relationship between school food and nutrition policies, suggesting that better supporting their implementation could increase the likelihood of their success.

## 1. Introduction

Supporting the implementation of strategies to encourage healthier eating is an international priority to address poor diet quality among children and youth [1]. Schools are an important intervention setting to promote childhood nutrition, and many jurisdictions have adopted school food and nutrition policies (SFNPs), guidelines and programs to modify the school nutrition environment and promote healthier eating [2,3]. Nutrition policies can help to create healthier school environments by influencing the availability of food and beverages, which subsequently may impact the nutrition behaviours of students [4,5,6,7,8,9]. A recent systematic review suggested that school policies have a positive effect on behavioural risk factors for non-communicable diseases (NCD), particularly when they are implemented as part of a comprehensive approach [10]. For example, policies aimed at reducing sugar-sweetened beverage intake or increasing fruit and vegetable intake in schools had corresponding impacts on consumption patterns, although findings were mixed for other NCD risk factors [10]. The authors noted that greater consideration of environmental or structural factors that help or hinder individual behaviours might offer a more equitable approach to policy implementation [10]. Thus, for policy implementation to affect the degree of change necessary for a sustained impact, there is a need to identify specific aspects of the school environment that will best support sustainable positive changes to childhood nutrition in school settings [10,11,12].

The east coast province of Nova Scotia (NS), Canada, has a rich history of policy action to support children’s health in schools. In 2006, the province was one of the first in Canada to launch an SFNP providing standards for foods and beverages served and sold in schools across the province [13]. These mandated standards included directives for school eating practices such as pricing, programming and advertising, and guidelines that encourage schools to foster community partnerships and support local food products [13]. Since the policy was introduced, funding has been distributed each year to support implementation in schools, but there are gaps in the implementation of directives that limit their potential for impact [14]. One such gap is in the ability for each school to implement policy directives based on adequacy of facilities or equipment and capacity of staff to do so. In a recent scoping review [15], we noted the complexity of real-world food policy implementation in schools and the importance of adequate recognition of school and community characteristics as influential to SFNP implementation. The purpose of this study was to explore the association of the combined factors of perceived adequacy and capacity for policy implementation with food availability and policy adherence in schools across NS. It was hypothesized that schools with greater perceived adequacy of facilities or equipment and capacity of staff to support policy implementation would be more able to adhere to the SFNP and to serve healthier foods.

## 2. Materials and Methods

A cross-sectional study was conducted in 2014–2015 to provide an assessment of policy implementation and adherence across NS, as they relate to the directives of the 2006 policy. An online survey was developed and administered to assess the implementation of the nutrition policy across all public schools in NS (elementary, junior and senior high). The online survey was hosted on a secure web-based platform from November 2014 to February 2015 and took about 15 min to complete. Following institutional review board approval and with permission and support from each school board key contact, school principals were contacted by email to request their participation in the online survey. The process (by research team or school board) and timing for contacting school principals were determined through the advice of our key school board contacts. Principals were instructed that they could also identify an appropriate designate with experience in school food service to complete the survey on behalf of the school. Two reminders to complete the survey were sent via email, and the survey link was also shared through social media.

The measures in the survey were based on the psychometric properties of scales from similar research conducted in Canada [16] and comprised questions related to the school food environment. These included organizational factors, school climate, policy institutionalization, perceived adequacy of facilities or equipment and capacity of staff to support policy implementation. The survey for this study added questions pertaining to the directives and guidelines of the NS school nutrition policy (available from the authors by request). Content review of the measures was completed by government stakeholders to determine the relevance of constructs and measures for the NS context.

The perceived adequacy of facilities or equipment and capacity of staff to support policy implementation represented a composite measure based on two dimensions that were self-reported by survey participants. These perceived adequacy and capacity constructs were derived from questions related to staffing, facilities and equipment available for food preparation when compared to other schools. Responses to these questions were then characterized as ‘below average’, ‘average’ or ‘above average’ in relation to perceived adequacy and capacity. Adherence to a breakfast, snack and lunch policy was self-reported through the availability of certain foods that were classified into food service types, that reflected foods that are likely to be ‘quick’ and ‘slow’ to prepare. Policy adherence was framed through asking ‘*To the best of your knowledge, to what extent are minimum nutrition food and beverages sold or served in*…’. Policy adherence for each type of school policy was then dichotomized to reflect that ‘no minimum nutrition foods served’ represented ‘policy adherence’.

Food service was then assessed within each school by asking *‘How often are the following foods and beverages served or sold from the school cafeteria, vending machines(s), snack bar or school store during school hours?*’. Food availability for each food was dichotomized as any frequency (‘daily’, 3–4 times per week, 1–2 times per week, 1–3 times per month or less than once per month’) or ‘never’. In order to select relevant foods served within schools, we conducted an exploratory factor analysis on a selection of policy-relevant foods. We extracted a two-factor solution using principal component analysis with promax rotation. The first component included nachos and poutine, garlic fingers, hamburgers and French fries which we labeled as ‘quick-service foods’; the second component included prepared fresh fruit, cooked or raw vegetables, sandwiches and subs, baked chicken or baked pasta dishes, which we labeled as ‘slow-service foods’. The two-factor solution explained 65% of the variance, and each scale had adequate internal consistency (Cronbach alpha) with alpha = 0.89 and 0.78, respectively. Slow- and quick-service food composition measures were dichotomized as foods served at any frequency (i.e., food available) or never serving one of the included foods.

In terms of covariates, self-reported survey questions were used to assess school grades, number of students and number of staff within each school. An indicator of community socioeconomic status (SES) was calculated from the median community income using 2016 census data and then matched with the school community name to create a proxy measure of schools. School rurality was assessed using the second character of school postal codes (0 representing rural, 1 representing urban). Descriptive statistics were used to summarize school characteristics, the combined measure of perceived adequacy and capacity for policy implementation, policy adherence, and food availability (both individual foods and composite measures) across school grades. Binary logistic regressions were first used to evaluate breakfast, snack and lunch policy adherence by level of perceived staffing and facility adequacy and capacity (unadjusted). Models were then adjusted for school size, community median income and rurality. Binary logistic regression was also used to evaluate slow- and quick-service food availability composite measures by adherence to breakfast, snack and lunch policy (unadjusted). Models were then adjusted for school size, community median income and rurality. Complete case analysis was used for missing outcome data, while missing exposure or covariate values were examined, with no significant differences in percentages across exposure levels or outcomes. Missing values were categorized for each variable and included in appropriate models to avoid additional case deletion (i.e., missing indicator approach). Analyses were conducted using Stata 12.

## 3. Results

Our sample included 237 schools across Nova Scotia, Canada (59% of all schools). Several schools comprised more than one grade level, and these included 170 elementary grades (primary/kindergarten to grade 6, ages 5–11 years), 85 junior high grades (grades 7 to 9, ages 12–14 years) and 56 high school grades (grades 10 to 12, ages 15–18 years) with an average of 332 students, and 33 staff per school (Table 1). Median community income across school locations in our sample was $30,627 CDN compared with $31,629 provincially, while 63% of schools in our sample were located in urban areas (versus 57% provincially). Adequacy of facilities or equipment and capacity of staff to support policy implementation were mostly reported as average; in some instances, greater percentages were below rather than above average. Staffing resources were reported as above average by 8.4%, below average by 24% and average by 59% of schools. Facility resources were reported as above average by 28%, below average by 22% and average by 43% of schools. Twenty-five percent of schools reported breakfast policy adherence, while snack policy adherence was reported by 22%, and lunch policy adherence was reported by 19% of schools. Quick-service foods were served in 64% of schools, while slow-service foods were served in 89% of schools.

The results of the regression analysis showed no association between staffing resources and policy adherence. However, schools reporting above-average facility resources were associated with greater odds of adhering to a school lunch policy after adjustment (OR = 3.62, CI = 1.56, 8.40) but not to a snack or breakfast policy (Table 2).

Additional results showed no association between adherence to a breakfast, snack or lunch policy and slow-service foods. However, schools that reported adherence to a snack and lunch policy were associated with lower odds of having quick-service foods available within the school after adjustment (OR = 0.48, CI = 0.23, 1.01 and OR = 0.18, CI = 0.08, 0.41, respectively, Table 3).

## 4. Discussion

This study sought to explore the association between adequacy of facilities or equipment and capacity of staff to support policy implementation with food availability and policy adherence in NS. It was hypothesized that schools with greater adequacy and capacity for policy implementation and adherence to the school nutrition policy would be more likely to serve healthier foods. This hypothesis was borne out in our results in certain circumstances, which suggested that schools with above-average facilities had more than three times (3.62) greater odds of adhering to a lunch policy, while schools that adhered to a snack and lunch policy had 52% and 82% lower odds of serving quick-service foods. The consistency of our findings with the results of a recent scoping review in the international literature [15], further reinforces the need to pay sufficient attention to policy implementation across multiple levels of the school food system and to ensure the engagement of all key stakeholders necessary to support policy directives. This review identified how the availability of resources, such as lack of adequate funding, school facilities, and resources, all impacted school food policy implementation internationally. It also highlighted the need for high-level direction, resources, infrastructure and administrative systems to support SFNP implementation [15]. Our study helps to quantify the magnitude of association for reported adequacy of facilities or equipment and capacity of staff to support policy implementation at a provincial level within Canada. However, the results also suggest that there are differential associations based on specific types of food availability and policy adherence, that may also differ across provinces and country contexts.

Following an exploratory factor analysis, this study considered two types of foods, ‘quick’- versus ‘slow’-service foods, as a proxy for the healthfulness of the types of foods available in schools. To our knowledge, this is the first data-driven use of this type of conceptualization for foods available in schools. Research has previously considered the impact of less healthy foods on the diets of children using terms such as ‘convenience or commercially prepared foods’ [17] or ‘fast-food’ [18]. One study examined the effect of fast-food and full-service restaurant consumption among children and youth and found that both were associated with higher energy intake and better diet quality [19]. Although ‘slow’-service foods may be considered intuitively healthier, further research is needed to determine how these—and ‘quick’-service foods—are associated with children’s diet quality.

Our finding that schools with well-equipped facilities were more likely to adhere to the school nutrition policy for lunch programs suggests that improvements to the physical infrastructure of schools may be necessary to ensure access to proper equipment to prepare healthier foods for students. Alternatively, these schools might simply have a more structured approach to policy implementation as a function of being well equipped. These differences may be particularly important for schools within communities of lower socioeconomic status, as research has found that these schools struggle with the resources required for policy implementation [20,21,22,23,24,25,26], whereas schools in communities with higher socioeconomic status can have more resources and opportunities and be better able to implement nutrition policies [27,28,29,30].

A strength of this study is the use of a data-driven approach that builds on the evidence from the aforementioned qualitative studies. The sample of schools, representing 59% of all schools in the province, was also large. There are some limitations, however. First is the use of self-reported data in assessing the school food environment. Self-report is known to be subject to bias and in this context, may lead to optimism bias, whereby the foods provided in schools were considered to be healthier than when assessed using objective measures. Second, our classification of foods as ‘slow’- or ‘quick’-service, while data-driven, may also not fully align with other examples from dietary pattern analyses, thereby limiting comparisons with other studies [31]. Third, we derived a proxy measure for school-level socioeconomic status based on median community income. While this is a suitable indicator to adjust for variation in community level income, it does not represent a direct measure of the actual resources available to schools, limiting our ability to look at modifications of these associations using a meaningful measure of socioeconomic status. A more sensitive measure of school-level SES should be considered in future research, as has been reported in research from the United States, through the use of school size, racial/ethnic composition and eligibility for free school meals as proxy measures for SES [32]. In addition, while our response rate was adequate for school-based research, this should be considered during the interpretation of the results. As reported in the results, the analytic sample and full sample of schools in Nova Scotia were similar across community-level SES and percentage of urban versus rural schools.

## 5. Conclusions

School nutrition policies have the potential to encourage healthier eating among children and youth. Our findings highlight the relationship between SFNPs and school adequacy of facilities or equipment and capacity of staff to support important aspects of policy adherence and food availability. Understanding the potential impact of these school-level factors on policy implementation helps to identify opportunities for intervention to support sustainable positive changes to childhood nutrition.

## Figures and Tables

**Table 1:** School characteristics for analytical samples by grades within each school, *N* = 237 ^1^.

Classification	Characteristic	School Grades			Total ^1^
		Elementary School	Jr. High School	High School	
Socio-demographic					
School	Number of school grades	170	85	56	311
	Mean number of students (SE)	268 (14)	340 (19)	537 (19)	332 (16)
	Mean number of staff (SE)	29.8 (1.5)	36.0 (1.7)	47.9 (3.3)	33.4 (1.2)
Community SES	Median community income (IQR)	$30,627 (7130)	$29,973 (7223)	$28,968 (5687)	$30,627 (7130)
School rurality	Urban (N)	58% (99)	55% (47)	50% (28)	63% (150)
Nutritional Resources					
Staffing	Average (N)	60% (102)	40% (57)	57% (32)	59% (141)
	Below average (N)	24% (41)	18% (15)	27% (15)	24% (57)
	Above average (N)	7.0% (12)	7.0% (6)	14% (8)	8.4% (20)
	Missing (N)				8.0% (19)
Facilities	Average (N)	44% (75)	41% (35)	41% (23)	43% (103)
	Below average (N)	24% (41)	22% (19)	14% (8)	22% (53)
	Above average (N)	25% (42)	31% (26)	43% (24)	28% (67)
	Missing (N)				5.9% (14)
Policy Adherence ^2^					
Breakfast	No minimum nutrition (N)	27% (47)	22% (19)	16% (9)	26% (61)
	At least some (N)	63% (107)	65% (55)	79% (44)	63% (149)
	Missing (N)				11% (27)
Snack	No minimum nutrition (N)	24% (42)	14% (12)	11% (6)	22% (53)
	At least some (N)	62% (106)	72% (61)	79% (44)	63% (150)
	Missing (N)				14% (34)
Lunch	No minimum nutrition (N)	22% (37)	14% (12)	13% (7)	19% (45)
	At least some (N)	69% (117)	71% (60)	82% (46)	70% (165)
	Missing (N)				11% (27)
Food availability ^3^					
Quick-service foods	Nacho and poutine (N)	22% (37)	42% (36)	46% (26)	26% (63)
	Never (N)	72% (122)	47% (40)	48% (27)	65% (154)
	Missing (N)				8% (20)
	Garlic fingers (N)	33% (57)	42% (36)	54% (30)	34% (81)
	Never (N)	60% (102)	46% (39)	41% (23)	57% (136)
	Missing (N)				8% (20)
	Hamburger (N)	48% (81)	65% (55)	73% (41)	52% (123)
	Never (N)	46% (78)	25% (21)	21% (12)	40% (94)
	Missing (N)				8% (20)
	French fries (N)	48% (82)	62% (53)	77% (43)	53% (126)
	Never (N)	44% (75)	25% (21)	14% (8)	37% (88)
	Missing (N)				10% (23)
Quick-service foods composite					
	Quick foods served (N)	59% (101)	73% (62)	88% (49)	64% (151)
	Never (N)	35% (59)	16% (14)	7% (4)	28% (67)
	Missing (N)				8% (19)
Slow-service foods	Fresh fruit (N)	79% (135)	79% (67)	91% (51)	80% (188)
	Never (N)	15% (25)	12% (10)	5% (3)	13% (31)
	Missing (N)				7% (18)
	Cooked/raw vegetables (N)	74% (126)	78% (66)	91% (51)	75% (176)
	Never (N)	20% (35)	14% (12)	5% (3)	19% (44)
	Missing (N)				7% (17)
	Sandwiches and subs (N)	73% (125)	74% (63)	89% (50)	73% (175)
	Never (N)	20% (35)	15% (13)	4% (2)	18% (43)
	Missing (N)				8% (19)
	Baked chicken (N)	62% (106)	66% (56)	77% (44)	62% (148)
	Never (N)	30% (52)	22% (19)	16% (9)	28% (67)
	Missing (N)				9% (22)
	Baked pasta dish (N)	166% (112)	72% (61)	91% (51)	68% (163)
	Never (N)	26% (45)	16% (14)	5% (3)	22% (53)
	Missing (N)				9% (21)
Slow-service foods composite					
	Slow foods served (N)	92% (156)	87% (74)	96% (54)	89% (211)
	Never (N)	4% (7)	5% (4)	0% (0)	5% (11)
	Missing (N)				6% (15)

SES = socioeconomic status, N = number, SE = Standard Error; IQR = Inter-Quartile Range; ^1^ Several schools are combined, therefore the number of school grades will be greater than the total number of schools; ^2^ Minimum nutrition foods served ‘never’; ^3^ Food availability is % of schools that serve foods more than ‘never’; composite measures are for serving any of the quick or slow foods and include missing data in totals.

**Table 2:** Odds ratios and 95% confidence intervals for breakfast, snack and lunch policy adherence by level of perceived nutritional resources including adequate staffing and facilities.

Odds of Policy Adherence ^1^	Level of Perceived Nutritional Resources		
	Average	Above Average	Below Average
Staffing			
Breakfast policy (*n* = 199)			
Unadjusted	REF	0.88 [0.43, 1.82]	0.97 [0.32, 2.92]
Adjusted ^2^	REF	0.87 [0.42, 1.82]	0.87 [0.28, 2.66]
Snack policy (*n* = 192)			
Unadjusted	REF	1.00 [0.47, 2.11]	1.80 [0.60, 5.36]
Adjusted ^2^	REF	0.83 [0.38, 1.82]	1.85 [0.57, 5.99]
Lunch policy (*n* = 196)			
Unadjusted	REF	1.43 [0.66, 3.09]	1.32 [0.40, 4.41]
Adjusted ^2^	REF	1.36 [0.62, 2.96]	1.44 [0.42, 4.95]
Facilities			
Breakfast policy (*n* = 203)			
Unadjusted	REF	1.12 [0.51, 2.47]	0.96 [0.47, 1.95]
Adjusted ^2^	REF	1.15 [0.52, 2.56]	0.99 [0.46, 2.13]
Snack policy (*n* = 197)			
Unadjusted	REF	1.68 [0.76, 3.70]	0.82 [0.38, 1.77]
Adjusted ^2^	REF	1.49 [0.66, 3.35]	1.26 [0.54, 2.91]
Lunch policy (*n* = 201)			
Unadjusted	REF	3.78 *** [1.64, 8.71]	1.36 [0.58, 3.18]
Adjusted ^2^	REF	3.62 *** [1.56, 8.40]	1.51 [0.62, 3.69]

^1^ No minimum nutrition foods served; ^2^ Adjusted for school size, community median income and rurality; *** *p* < 0.001.

**Table 3:** Odds ratios and 95% confidence intervals for slow- and quick-service food availability by breakfast, snack and lunch policy adherence.

Odds of Food Availability ^1^	Do not Adhere to Policy ^2^	Adhere to Breakfast Policy	Adhere to Snack Policy	Adhere to Lunch Policy
Slow-service foods (*n* = 208)				
Unadjusted	REF	2.93 [0.35, 24.33]	0.58 [0.13, 2.51]	0.82 [0.16, 4.22]
Adjusted ^3^	REF	3.04 [0.35, 26.28]	0.77 [0.16, 3.69]	1.13 [0.21, 6.15]
Quick-service foods (*n* = 205)				
Unadjusted	REF	0.74 [0.38, 1.41]	0.37 *** [0.19, 0.72]	0.20 *** [0.10, 0.40]
Adjusted ^3^	REF	0.76 [0.36, 1.59]	0.48 * [0.23, 1.01]	0.18 *** [0.08, 0.41]

^1^ Slow or fast foods provided daily, weekly or monthly within the school; ^2^ No minimum nutrition foods served = policy adherence; ^3^ Adjusted for school size, neighbourhood median income and rurality; * *p* < 0.05, *** *p* < 0.001.

## References

[1] World Health Organization (2003). Diet, Nutrition and the Prevention of Chronic Diseases.

[2] Blanck H.M., Kim S.A. (2012). Creating supportive nutrition environments for population health impact and health equity: An overview of the Nutrition and Obesity Policy Research and Evaluation Network’s efforts. Am. J. Prev. Med..

[3] Story M., Kaphingst K.M., Robinson-O’Brien R., Glanz K. (2008). Creating healthy food and eating environments: Policy and environmental approaches. Annu. Rev. Public Health.

[4] Probart C., McDonnell E.T., Jomaa L., Fekete V. (2010). Lessons from Pennsylvania’s mixed response to federal school wellness law. Health Aff. Proj. Hope.

[5] Brener N.D., Chriqui J.F., O’Toole T.P., Schwartz M.B., McManus T. (2011). Establishing a Baseline Measure of School Wellness-Related Policies Implemented in a Nationally Representative Sample of School Districts. J. Am. Diet. Assoc..

[6] Evenson K.R., Ballard K., Lee G., Ammerman A. (2009). Implementation of a school-based state policy to increase physical activity. J. Sch. Health.

[7] Kelder S.H., Springer A.S., Barroso C.S., Smith C.L., Sanchez E., Ranjit N., Hoelscher D.M. (2009). Implementation of Texas Senate Bill 19 to Increase Physical Activity in Elementary Schools. J. Public Health Policy.

[8] Barroso C.S., Kelder S.H., Springer A.E., Smith C.L., Ranjit N., Ledingham C., Hoelscher D.M. (2009). Senate Bill 42: Implementation and impact on physical activity in middle schools. J. Adolesc. Health Off. Publ. Soc. Adolesc. Med..

[9] Chriqui J.F., Pickel M., Story M. (2014). Influence of school competitive food and beverage policies on obesity, consumption, and availability: A systematic review. JAMA Pediatr..

[10] Singh A., Bassi S., Nazar G.P., Saluja K., Park M.H., Kinra S., Arora M. (2017). Impact of school policies on non-communicable disease risk factors—A systematic review. BMC Public Health.

[11] Taylor J.P., McKenna M.L., Butler G.P. (2010). Monitoring and evaluating school nutrition and physical activity policies. Can. J. Public Health Rev. Can. Santé Publique.

[12] Kumanyika S.K. (2014). Five Critical Challenges for Public Health. Health Educ. Behav..

[13] Province of Nova Scotia (2008). Food and Nutrition Policy Documents. Food and Nutrition in Nova Scotia Schools. https://www.ednet.ns.ca/docs/foodnutritionpolicyguidelines.pdf.

[14] McIsaac J.-L.D., Chu Y.L., Blanchard C., Rossiter M., Williams P., Raine K., Kirk S.F., Veugelers P.J. (2015). The impact of school policies and practices on students’ diets, physical activity levels and body weights. A province-wide practice-based evaluation. Can. J. Public Health..

[15] McIsaac J.L., Spencer R., Meisner K., Kontak J., Kirk S.F.L. (2018). Factors influencing the implementation of nutrition policies in schools: A scoping review. Health Educ. Behav..

[16] Mâsse L.C., de Niet J.E. (2013). School nutritional capacity, resources and practices are associated with availability of food/beverage items in schools. Int. J. Behav. Nutr. Phys. Act..

[17] Alexy U., Libuda L., Mersmann S., Kersting M. (2011). Convenience foods in children’s diet and association with dietary quality and body weight status. Eur. J. Clin. Nutr..

[18] Braithwaite I., Stewart A.W., Hancox R.J., Beasley R., Murphy R., Mitchell E.A., ISAAC Phase Three Study Group (2014). Fast-food consumption and body mass index in children and adolescents: An international cross-sectional study. BMJ Open.

[19] Powell L.M., Nguyen B.T. (2013). Fast-food and full-service restaurant consumption among children and adolescents: Effect on energy, beverage, and nutrient intake. JAMA Pediatr..

[20] Aarestrup A.K., Jørgensen T.S., Jørgensen S.E., Hoelscher D.M., Due P., Krølner R. (2015). Implementation of strategies to increase adolescents’ access to fruit and vegetables at school: Process evaluation findings from the Boost study. BMC Public Health.

[21] Bauer K.W., Larson N.I., Nelson M.C., Story M., Neumark-Sztainer D. (2009). Socio-environmental, personal and behavioural predictors of fast-food intake among adolescents. Public Health Nutr..

[22] Downs S.M., Farmer A., Quintanilha M., Berry T.R., Mager D.R., Willows N.D., McCargar L.J. (2012). From Paper to Practice: Barriers to Adopting Nutrition Guidelines in Schools. J. Nutr. Educ. Behav..

[23] Mâsse L.C., Naiman D., Naylor P.-J. (2013). From policy to practice: Implementation of physical activity and food policies in schools. Int. J. Behav. Nutr. Phys. Act..

[24] Nanney M.S., Davey C.S., Kubik M.Y. (2013). Rural Disparities in the Distribution of Policies that Support Healthy Eating in US Secondary Schools. J. Acad. Nutr. Diet..

[25] Nathan N., Wolfenden L., Butler M., Bell A.C., Wyse R., Campbell E., Milat A.J., Wiggers J. (2011). Vegetable and fruit breaks in Australian primary schools: Prevalence, attitudes, barriers and implementation strategies. Health Educ. Res..

[26] Phillips M.M., Goodell M., Raczynski J.M., Philyaw Perez A.G. (2012). Creating and Using Index Scores in the Analysis of School Policy Implementation and Impact. J. Sch. Health.

[27] Walton M., Waiti J., Signal L., Thomson G. (2010). Identifying barriers to promoting healthy nutrition in New Zealand primary schools. Health Educ. J..

[28] Vine M.M., Elliott S.J. (2014). Examining local-level factors shaping school nutrition policy implementation in Ontario, Canada. Public Health Nutr..

[29] Vine M.M., Elliott S.J., Raine K.D. (2014). Exploring Implementation of the Ontario School Food and Beverage Policy at the Secondary-School Level: A Qualitative Study. Can. J. Diet. Pract. Res..

[30] Gregorič M., Pograjc L., Pavlovec A., Simčič M., Gabrijelčič Blenkuš M. (2015). School nutrition guidelines: Overview of the implementation and evaluation. Public Health Nutr..

[31] Hodge A., Basset J. (2016). Editorial what can we learn from dietary pattern analysis?. Public Health Nutr..

[32] Sanchez-Vaznaugh E.V., Sánchez B.N., Crawford P.D., Egerter S. (2015). Association Between Competitive Food and Beverage Policies in Elementary Schools and Childhood Overweight/Obesity Trends Differences by Neighborhood Socioeconomic Resources. JAMA Pediatr..

